# Plasma Kallikrein Cleaved H-kininogen: An End-Point Marker for Contact Activation *in vitro* and *ex vivo*

**DOI:** 10.3389/fcvm.2022.873975

**Published:** 2022-05-20

**Authors:** Yaseelan Palarasah, Stephanie Thuy Duong Pham, Jørgen Brodersen Gram, Jonas Heilskov Graversen, Katrine Pilely, Johannes Jakobsen Sidelmann

**Affiliations:** ^1^Unit for Thrombosis Research, Department of Regional Health Research, University of Southern Denmark, Esbjerg, Denmark; ^2^Department of Clinical Biochemistry, University Hospital of Southern Denmark, Esbjerg, Denmark; ^3^Department of Cancer and Inflammation Research, Institute for Molecular Medicine, University of Southern Denmark, Odense, Denmark

**Keywords:** contact system, specific antibody, cleaved H-kininogen, ELISA, reference interval, blood contacting biomaterials

## Abstract

**Objectives:**

The contact system consists of coagulation factor XII (FXII), prekallikrein, and H-kininogen (HK) and plays important roles in many diseases. Plasma kallikrein (PKa) cleaved HK (cHK) is a marker of contact activation. Presently, we developed a specific and precise enzyme-linked immunosorbent assay (ELISA) for determination of cHK *in vitro* and *ex vivo.*

**Methods:**

Cleaved HK specific mouse monoclonal antibodies (mAbs) were generated using a peptide corresponding to the PKa cleavage site on HK as immunogen. ELISA, surface plasmon resonance analysis, and immunoprecipitation established the specificity of the antibody, which subsequently was used in a sandwich ELISA. The analytical imprecision and the concentration of cHK in a reference population and in women receiving oral contraceptives (OC) were determined. cHK was assessed *in vitro* in plasma exposed to polytetrafluoroethylene, silicone, and glass tubes.

**Results:**

The selected mAb showed excellent specificity towards cHK. The intra-assay and inter-assay CV of the ELISA were 3.6 and 6.0%, respectively. The reference population (60 women, 60 men) displayed a median cHK plasma concentration of 1.38 μg/mL and a reference interval of 0.82 – 2.56 μg/mL. Women receiving OC had significantly higher concentrations, *p* < 0.001. cHK was significantly elevated in plasma exposed to polytetrafluoroethylene, *p* = 0.001, and glass, *p* < 0.0001.

**Conclusion:**

The ELISA showed excellent precision and specificity. cHK assessment *ex vivo* demonstrated ongoing contact activation in healthy individuals, augmented by OC. The cHK antibody and the ELISA could be promising tools in contact activation related diseases and *in vitro* investigations of the plasma compatibility of blood contacting biomaterials.

## Introduction

The contact system consists of the plasma proteins coagulation factor XII (FXII), prekallikrein (PK), and H-kininogen (HK). Activation of the contact system *in vivo* leads to release of the end-product bradykinin (BK) from HK. The system plays important roles in inflammation, sepsis, cancer, coagulation, and fibrinolysis (1). Misfolded proteins, polyphosphate, exposed vessel wall collagen, DNA/RNA fragments and inborn enzymatic activity of FXII and PK are all of importance for activation of the contact system (2, 3).

*In vitro* activation of the contact system is prominently observed in relation to blood contacting biomaterials (BCB) such as blood collection tubes, but also stents, indwelling catheters, and extracorporeal and dialysis devices may activate the contact system. The interactions between BCB, FXII, PK, and coagulation factor FXI (FXI) result in initiation and activation of the contact driven pathway of coagulation, culminating in thrombin generation and fibrin polymerization. Serious thrombotic complications may follow the use of BCB, and despite more than 50 years of research, a genuine blood compatible artificial device still eludes. This has been described as the “blood compatibility catastrophe of the 21st century” (4). Recent studies suggest an important role of the contact system in BCB driven thrombosis (5). Thus, there is a need for specific and sensitive methods for evaluation of *ex vivo* and *in vitro* activation of the contact system.

Current methods for measuring contact activation are semi-quantitative or characterized by low specificity and low sensitivity in a range of clinical conditions. The approaches include measurements of BK and breakdown products of BK using mass spectrometry (6), cleaved/BK-free HK assessed by western blot (7), FXIIa activity (8), and quantification of complexes between plasma kallikrein (PKa) or FXIIa and complement C1 esterase inhibitor (C1inh) (9). Short half-life, instability of the analyte, and insufficient assay sensitivity hamper the validity of these measures of contact activation (10, 11). Fast-form α_2_-macroglobulin (F-α_2_M) may be a promising indicator of contact activation *in vitro* as previously reported (12).

In the present study, we have focused on cleaved HK (cHK) as end-point marker for contact activation. HK is a liver synthesized single-chain glycoprotein with a molecular weight of approximately 120 kDa (13). The plasma concentration is 70–90 μg/mL (14). HK consists of the six domains: D1-D6. The domains D1-D3 are the heavy chain of HK, D4 contains the peptide bradykinin, and the domains D5-D6 compose the light chain of HK (15, 16). The domains D2 and D3 function as inhibitors of cysteine and platelet-binding activity (17, 18). The D1 domain lacks inhibitory properties (17) but harbors a low-affinity calcium-binding site (19). Domain D5 is responsible for mediating the binding of HK to negatively charged surfaces, while domain D6 holds the binding sites for both PK(a) and FXI (15, 20). PKa has the capacity to cleave HK leading to formation of BK and cHK. PKa preferentially cleaves HK (21) but also tissue kallikrein may cleave HK although with a different cleavage site than PKa (22).

Presently we report on the evolution of specific monoclonal antibodies (mAbs) directed against the PKa cleavage site on HK. Selected cHK specific mAbs were characterized and used to establish a cHK specific ELISA with sufficient sensitivity to quantify contact activation both *ex vivo* and *in vitro*.

## Materials and Methods

### Buffers, Solutions, and Proteins

Coating buffer: 15 mM Na_2_CO_3_, 35 mM NaHCO_3_, pH 9.6. PBS: 1.45 mM NaH_2_PO_4_, 6.46 mM Na_2_HPO_4_, 2.7 mM KCl, 137 mM NaCl, pH 7.4. PBS-TW: PBS, 0.05% Tween-20, pH 7.4. Blocking buffer: PBS-TW, 0.5% skim milk powder (Honeywell Fluka™, Charlotte, NC, United States). Dilution buffer: PBS-TW, 0.06% skim milk powder, 100 KIU/mL aprotinin (Trasylol^®^ 10.000 KIU/mL, Nordic Group B.V., Hoofddorp, Netherlands), PBS-HSA: PBS, 0.1% human serum albumin (HSA). Acetate buffer: 50 mM CH_3_COOH, 33.75 mM NaOH, pH 5.0. Carbozole staining solution: 0.04% 3-amino-9-ethylcarbazole, 0.015% H_2_O_2_. Running buffer: 10 mM Hepes, 150 mM NaCl, and 0.05% Tween 20, pH 7.4. Dextran sulfate (100 μg/mL in H_2_O) with a molecular weight of 500 kDa (Pharmacia AB, Uppsala, Sweden). Peptide solutions (4 mg/mL) C-MISLMK and C-QPLGMISLMKRPPGFSPFRSSRIGEIKEE in 137 mM NaCl, purity ≥ 85% were purchased from Genscript (Piscataway, New Jersey, United States). Purified HK and cHK (1 mg/vial) were obtained from Enzyme Research Laboratories, South Bend, IN, United States.

### Generation of Human Cleaved H-kininogen Specific Monoclonal Antibodies

**FIGURE 1:**
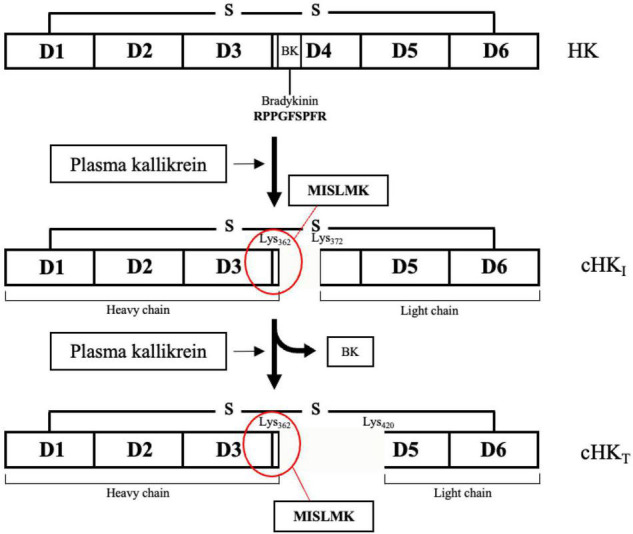
The domain structure of H-kininogen (HK). HK is a single-chain structure consisting of six domains (D1-D6). D1-D3 comprises the heavy chain. Motifs on D4, containing the peptide bradykinin, link the light chain, comprising D5-D6, to the heavy chain. BK is released from HK after cleavage of HK by primarily plasma kallikrein (PKa) yielding an intermediate cleaved HK (cHK_I_). After release of BK, the heavy and the light chain remain linked by a disulfide bond. A further cleavage of HK by PKa yields a truncated form of HK (cHK_T_). Figure modified from Zhang et al. (15).

NMRI mice were immunized with a synthetic peptide, consisting of an N-terminal Cys residue followed by a sequence representing the PKa cleavage site on HK, the six N-terminal residues prior to the bradykinin peptide of HK (C-MISLMK) (Figure 1). The mice were immunized twice subcutaneously, with an interval of at least 14 days, with 25 μg of the synthetic peptide coupled to diphtheria toxoid *via* the end cysteine and adsorbed to Al(OH)_3_ and mixed in a 1:1 ratio with Freund’s incomplete adjuvant. Fourteen days after the last injection and 3 days prior to the fusion, the mice received an intravenous boost with 12 μg of the coupled peptide.

Fusion of the isolated spleen cells with myeloma cells, to generate hybridoma cells and the selection procedure, was essentially conducted as described by Köhler and Milstein (23), except the SP2/0-AG14 myeloma cell line was selected as fusion partner. Positive hybridoma clones were selected using two different ELISA setups; screening against 0.5 μg/mL purified HK contra cHK in parallel with screening against the immunized peptide the C-MISLMK peptide contra the overlapping peptide C-QPLGMISLMKRPPGFSPFRSSRIGEIKEE, both conjugated with ovalbumin (Sigma-Aldrich, St. Louis, MO, United States). Positive hybridomas were identified and cloned by the limited dilution method until single clones were obtained. Single clones were then grown in culture flasks in RPMI-1640 (Lonza, BioWhittaker^®^, Basel, Switzerland) supplemented with sodium pyruvate (Gibco™, Waltham, MA, United States) and gentamicin sulfate (biowest^®^, Nuaillé, France) and containing 10% fetal bovine serum (biowest^®^). The mAbs were purified from culture supernatants by using Protein G affinity chromatography.

### Surface Plasmon Resonance Analysis

The surface plasmon resonance (SPR) analysis of binding of the mAbs to HK and cHK was carried out using a Biacore T200 instrument (Cytiva, Albertslund, Denmark). The sensor chips (type CM5) were activated with a 1:1 mixture of 0.2 M N-ethyl-N′-(3-dimethylaminopropyl) carbodiimide and 0.05 M N-hydroxysuccimide in H_2_O for 7 min. For screening of mAbs directed against HK and cHK, purified HK or cHK at 10 μg/mL in 10 mM sodium acetate, pH 5.0 were immobilized on the activated chip, and the remaining reactive sites were blocked with an 7 min exposure of the chip to 1 M ethanolamine, pH 8.5. The SPR signal, generated from immobilized HK/cHK, generally corresponded to 7–50 fmol of protein/mm^2^. The running buffer for SPR experiments was 150 mM NaCl, 10 mM HEPES, 0.05% Tween-20 pH 7.4. Sensorgrams were generated by injecting mAb concentrations in the range of 0.04–10 μg/mL. The flow cells were regenerated with 0.2M H_3_PO_4_.

In another setup, mAbs at 10 μg/mL were immobilized on the chip in 10 mM sodium acetate, pH 5.0. Addition of 1 M ethanolamine, pH 8.5 blocked the remaining binding sites. The SPR signal generated from immobilized mAb generally corresponded to 5–15 fmol of protein/mm^2^. Sensorgrams for HK/cHK binding were generated using the same running buffer as before and with HK/cHK concentrations in the range of 0.08–20 μg/mL. The flow cells were regenerated with 50 mM glycine, 1 mM EDTA, 0.5 mM NaCl, 0.05% Surfactant P-20, pH 2.5.

The Biacore evaluation software version 3.1 were used for evaluation of the binding data. All SPR experiments were performed in independent triplicates at least.

### Immunoprecipitation, SDS-PAGE, and Western Blotting

Immunoprecipitation was performed by incubation of 2 μg/mL of the mAb 19-20-3 or native mAb HK-6, generated as previously described (24), with Dynabeads^®^ M-280 Sheep anti-Mouse IgG (Invitrogen, Waltham, MA, United States) end-over-end for 60 min at 4°C. After three times of washing with PBS-HSA, the beads incubated end-over-end for 60 min at 4°C with normal human plasma pool (NHP) and with NHP activated with glass (NHP + Glass) (see the section “*In vitro* contact activation of normal human plasma”) to allow antigen binding. After washing steps in PBS-HSA, bound antigen was eluted with 0.5% citric acid and the samples, together with control samples of purified HK and cHK, were subjected to Sodium dodecyl sulfate–polyacrylamide gel electrophoresis (SDS-PAGE), followed by western blotting.

Sodium dodecyl sulfate–polyacrylamide gel electrophoresis was performed on NuPAGE^®^Novex^®^ 4–12% Bis-Tris Plus 1.0 mm×17 well in NuPAGE^®^ MES SDS running buffer and using the molecular weight marker Novex^®^ Sharp Unstained Protein Standard all from Invitrogen (Waltham, MA, United States).

Proteins were electro-blotted onto a polyvinylidene difluoride (PVDF) membrane (Mini Format, 0.2 μm PVDF) (Bio-Rad Laboratories. Inc., Hercules, CA, United States) using the Trans-Blot^®^Turbo™ Transfer system (Bio-Rad Laboratories). After blocking the membrane overnight at 4°C in PBS-TW, the membrane was incubated with biotinylated polyclonal (pAb) rabbit anti-HK (Abcam, Cambridge, England) in PBS-TW. After washing steps in PBS-TW the membrane incubated in HRP conjugated streptavidin (Invitrogen) diluted 1:3000 in PBS-TW at RT for 30 min. Finally, the membrane was developed with carbazole staining solution in acetate buffer after three washes in PBS-TW.

### Biotinylation of Antibodies

Polyclonal antibody rabbit anti-HK (Abcam) were biotinylated by first dialyzing against 0.1 M NaHCO_3_ and then adding (+)-Biotin N-hydroxysuccinimide ester (BNHS) (Sigma-Aldrich, St. Louis, MO, United States) in a 1:100 dilution to the total volume of the protein solution. The solution was gently mixed while incubating for 3 h at RT. Unbound biotin was removed by dialysis against PBS in a Spectra/Por^®^ Dialysis Membrane molecular weight cut-off (MWCO) 6–8 kDa (Spectrum Laboratories Inc., Rancho Dominquez, CA, United States).

### Cleaved H-kininogen Specific Sandwich Enzyme-Linked Immunosorbent Assay

The cHK assay was constructed as a non-competitive sandwich ELISA, using the mAb 19-20-3 as capture antibody. Biotinylated mAb 19-31-18, generated as previously described (24), was used as detection antibody. MaxiSorp plates (Thermo Fisher Scientific) were coated and incubated overnight at 4°C with 100 μL/well of 2 μg/mL mAb 19-20-3 diluted in PBS. The wells were washed three times in PBS-TW and blocked with PBS-TW/0.5% skim milk (Honeywell Fluka™) for 60 min at RT to block remaining reactive sites. The calibrator (see the section “Parallelism, calibration, and statistical analysis”) and samples diluted in dilution buffer were applied to the wells and incubated for 60 min at RT with constant agitation. Following another three washes in PBS-TW, 100 μL of biotinylated mAb 19-31-18 diluted 1:8000 in PBS-TW was added to each well. The plates were incubated for 60 min at RT with constant agitation, followed by three times of washing in PBS-TW and incubated with 100 μL of HRP conjugated streptavidin (Invitrogen) diluted 1:5000 in PBS-TW for another 60 min at RT with constant agitation. Lastly, the plates were washed three times in PBS-TW and incubated with 100 μL TMB-Ultra (Thermo Fisher Scientific) in each well for 10 min at RT in the dark. Addition of 100 μL/well of 0.2 M H_2_SO_4_ stopped the color development and the optical density (OD) was measured at 450 nm.

### *In vitro* Contact Activation of Normal Human Plasma

A pool of citrate plasma samples from 100 blood donors from the Blood Bank at Odense University Hospital, Odense, Denmark served as normal human plasma (NHP). Blood was collected in evacuated Hemogard 9NC tubes (Becton Dickinson, Plymouth, United Kingdom) containing sodium citrate (2.7 mL of blood and 0.3 mL of 0.109 mol/L trisodium citrate). Platelet-poor plasma was prepared by centrifugation at 2000*g* for 20 min within 1 h after sampling. The plasma was stored at −80°C until analysis.

Contact activation was induced by incubation of 50 μL NHP in glass tubes (ø10 × 75 mm) (KEBO LAB A/S, Albertslund, Denmark) or with a final concentration of 50 μg/mL dextran sulfate (DXS), added to a final volume of 100 μL in polypropylene tubes (1.5 mL, 10.8 × 39 mm) (Sarstedt AG & Co., KG, Nürnbrecht, Germany). The tubes incubated at 37°C for 30 min with constant agitation.

### Analysis of Intra- and Inter-Assay Variation and Limit of Detection

The intra-assay variation of the cHK assay was determined by testing one sample at one occasion in forty wells, while the inter-assay variation was determined by testing three samples at five different occasions. The mean values, standard deviation (SD), and the coefficient of variation (CV) were calculated for each of these setups. The limit of detection (LOD) of the cHK assay was defined as the cHK concentration corresponding to the mean of the background absorbance plus 3.3 times the SD.

### Parallelism, Calibration, and Statistical Analysis

Parallelism between dilutions of the calibrator and purified cHK was tested by comparing the OD obtained in purified cHK diluted in buffer and in kininogen depleted plasma (Affinity Biologicals^ TM^ Inc., Ancaster, ON, Canada) spiked with 250 ng/mL purified cHK, followed by a twofold serial dilution.

The cHK assay was calibrated against serial dilutions of kininogen depleted plasma (Affinity Biologicals™ Inc.) spiked with 250 ng/mL purified cHK. OD values were recorded at 450 nm to generate a 10-point calibration curve using four-parameter logistic fit model using Softmax Pro^®^ (Molecular Devices, San Jose, CA, United States). The calibrator and samples were analyzed in duplicates. GraphPad Prism 9 (GraphPad Software, San Diego, CA, United States) was used for data analysis. The distribution of results was evaluated by the Kolmogorov–Smirnov test. Between groups, comparisons were performed using the Mann–Whitney U-test. Parallelism was deduced by Deming Regression Analysis.

### Reference Interval and Samples From Women Receiving Oral Contraceptives

The reference interval for cHK was established according to the EP28 A3C guideline from Clinical Laboratory Standards Institute (25). Citrate stabilized plasma from 120 healthy blood donors (60 women and 60 men) served as reference material. Citrate stabilized blood was additionally collected from 26 healthy female blood donors subscribed to OC. The Blood Bank of the University Hospital of Southern Denmark, Esbjerg, Denmark provided the samples. Plasma was isolated by centrifugation at 2000*g* for 20 min. and stored in cryotubes (Sarstedt AG & Co, Nürnbrecht, Germany) at −80°C until analysis. The 5–95 percentile range of the distribution defined the reference range.

### Biomaterial Evaluation

Contact activation induced by silicone (VWR International A/S, Søborg, Denmark), polytetrafluoroethylene (PFTE) (VWR International A/S), and glass tubes (ø10 × 75 mm) (KEBO LAB A/S) was investigated *in vitro*. Silicone and PFTE tubings were cut into 3 cm pieces. Fifty μL of NHP was added to the pieces and to glass tubes and incubated for 2 h at 37°C. Each material was investigated 10 times. The plasma was subsequently diluted 1:100 in dilution buffer and analyzed using the cHK assay.

## Results

### Human Cleaved H-kininogen Specific mAbs

The specificity of the mAbs towards cHK was validated by screening the hybridoma cells against purified HK relative to cHK and against the immunized synthetic peptide C-MISLMK contra the peptide C-QPLGMISLMKRPPGFSPFRSSRIGEIKEE corresponding to the overlapping sequence of intact HK. The experiments were performed in order to identify the antibodies recognizing HK and the overlapping peptide, and to ensure selection of antibodies with specificity for cHK.

**FIGURE 2:**
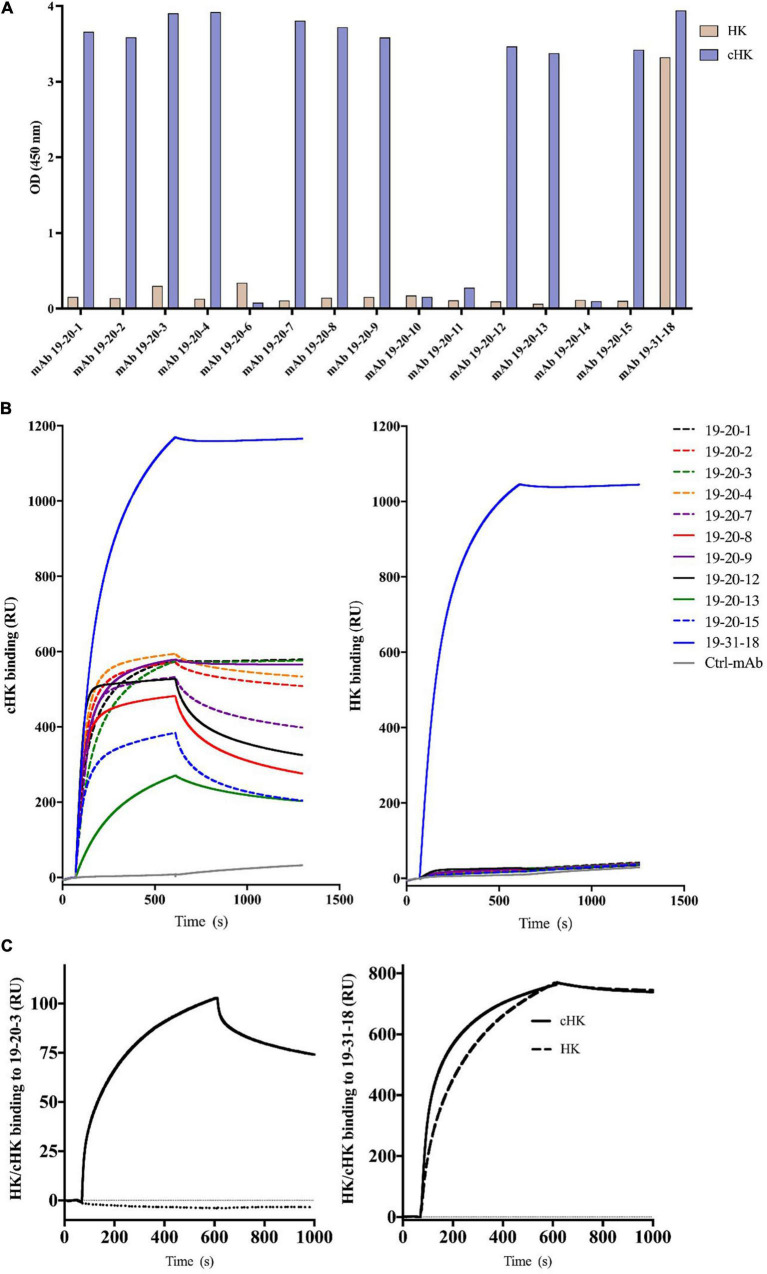
**(A)** Reactivity of cleaved H-kininogen (cHK) specific monoclonal antibodies (mAbs). ELISA analysis of the specificity of cHK mAbs toward cHK and HK: Microtiter plates were coated with 0.5 μg/mL purified HK or purified cHK. The mAb 19-31-18 reacting with both HK and cHK was used as a positive control. Two μg/mL of mAbs were added to the wells. Reactivity levels are given as optical density (OD) at 450 nm. **(B)** Surface plasmon resonance (SPR) analysis of binding of mAbs (5 μg/mL) to immobilized cHK and HK (35 pmol/mm^2^). Murine mAb against diphtheria toxoid was used as control (Ctrl-mAb). **(C)** SPR analysis of binding of HK/cHK (both 10 μg/mL) to immobilized mAbs: Binding of purified HK (dotted line) and cHK (solid line) to immobilized mAb 19-20-3 (left) and mAb 19-31-18 (right) (12 fmol of protein/mm^2^) was analyzed.

The panel of mAbs obtained from the screening towards cHK included mAb 19-20-1, mAb 19-20-2, mAb 19-20-3, mAb 19-20-4, mAb 19-20-7, mAb 19-20-8, mAb 19-20-9, mAb 19-20-12, mAb 19-20-13, and mAb 19-20-15 (Figure 2A). These mAbs were specific to the cleavage site exposed by PKa cleavage of HK. The antibodies bound only the immunized peptide (C-MISLMK) and not the overlapping peptide while binding to purified cHK but not HK (Figure 2A). The mAb 19-31-18 binding to both HK and cHK was used as a positive control.

### Evaluation of the Specificity of Human Cleaved H-kininogen Specific mAbs in Surface Plasmon Resonance Analysis

The binding to cHK by the panel of mAbs mentioned above and mAb 19-31-18 was investigated using SPR analysis. Several mAbs exhibited good binding to cHK, while positive control mAb 19-31-18 exhibited good binding to both cHK and HK (Figure 2B). All the cHK specific mAbs (19-20-x) generated a similar pattern to that of a negative control murine mAb, directed against diphtheria toxoid, indicated no binding to HK (Figure 2B).

Based on the initial SPR screening we selected mAb 19-20-3 for further SPR analysis. Monoclonal antibody 19-20-3 and mAb 19-31-18 were immobilized on the chip to elucidate a setup, without avidity contribution and the binding of cHK and HK to the immobilized mAbs was investigated. Both mAbs exhibited strong binding to cHK, albeit with a considerable stronger binding of 19-31-18. Only 19-31-18 exhibited binding to HK and exhibited near identical binding to HK and cHK. Representative sensorgrams are shown in Figure 2C and the determined binding parameters in Table 1. The binding parameters of mAb 19-20-3 and 19-31-18 to immobilized HK and cHK were also investigated. Both mAbs exhibited strong binding to cHK. Studying the binding at two different densities of immobilized cHK, it was demonstrated that the contribution from avidity to interaction in this experimental design was much more pronounced for 19-20-3 than for 19-31-18 (Table 2).

**TABLE 1:** Binding parameters of the interaction between immobilized monoclonal antibody (mAb) 19-20-3 and 19-31-18 (0.012 pmol/mm^2^) and H-kininogen (HK) or cleaved HK (cHK).

mAb on chip	Analyte	K_*D*_ (nM)	k_*a*_ (10^4^/MS)	k_*d*_ (10^–4^/s)
19-20-3	cHK	7.5 ± 2.1	4.5 ± 1.2	5.3 ± 1.3
	HK	n.a.	n.a.	n.a.
19-31-18	cHK	0.34 ± 0.10	8.4 ± 2.5	0.27 ± 0.01
	HK	0.35 ± 0.10	5.3 ± 0.1	0.19 ± 0.06

**TABLE 2:** Binding parameters of the interactions between immobilized H-kininogen (HK) or cleaved HK (cHK) and monoclonal antibody (mAb) 19-20-3 or 19-31-18.

Protein on chip	mAb	Chip density (fmol/mm^2^)	K_*D*_ (nM)	k_*a*_ (10^5^/MS)	k_*d*_ (10^–5^/s)
cHK	19-20-3	8	0.13 ± 0.01	9.2 ± 1.1	12.1 ± 1.6
		35	0.037 ± 0.02	7.7 ± 0.9	2.9 ± 0.9
cHK	19-31-18	8	0.028 ± 0.011	3.4 ± 1.4	0.95 ± 0.35
		35	0.019 ± 0.012	3.0 ± 0.1	0.54 ± 0.43
HK	19-31-18	32	0.028 ± 0.022	2.7 ± 0.2	0.73 ± 0.53

### Evaluation of the Specificity of Anti-human Cleaved H-kininogen mAbs in Immunoprecipitation, SDS-PAGE, and Western Blotting

**FIGURE 3:**
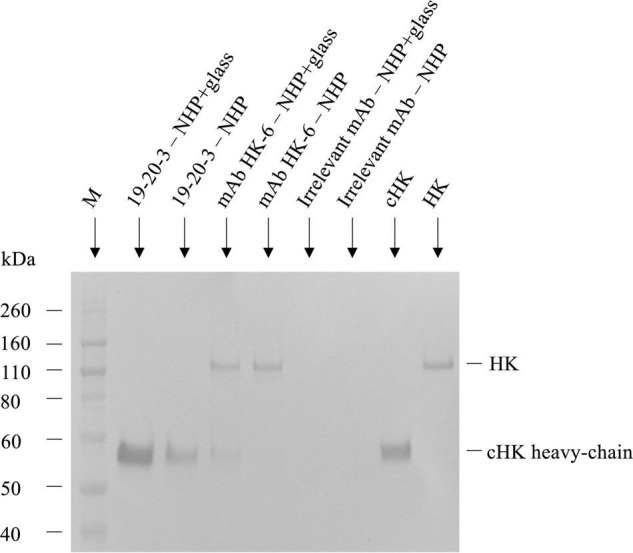
Reactivity of cleaved H-kininogen (cHK) specific monoclonal antibodies (mAbs) by immunoprecipitation. The reactivity of cHK specific mAb 19-20-3 and native HK mAb 19-31-6 were analyzed by immunoprecipitation followed by SDS-PAGE and western blotting. cHK and HK were immunoprecipitated by the mAbs from glass activated normal human plasma (NHP + glass) and normal human plasma (NHP). The samples were subjected to SDS-PAGE and western blotting, followed by development with biotinylated polyclonal rabbit anti-HK. Lane M indicate the molecular weight marker.

The reactivity of mAb 19-20-3 was further evaluated and compared with mAb HK-6 (24), reacting against native HK by immunoprecipitation followed by SDS-PAGE and western blotting, using biotinylated pAb rabbit anti-HK for detection and purified HK and cHK as controls (Figure 3). The immunoprecipitation, SDS-PAGE, and western blotting experiments showed, under reduced conditions, and using NHP + Glass as source, that mAb 19-20-3 precipitated a distinct band with a molecular mass of approximately ∼62 kDa, corresponding to the size of cHK heavy chain. A fainter band with a molecular mass of ∼62 kDa, corresponding to the cHK heavy chain, was precipitated, using NHP as source.

The immunoprecipitation and analysis by SDS-PAGE and western blotting under reduced conditions showed mAb HK-6, recognizing native HK and cHK, precipitated a ∼120 kDa band when NHP + Glass was used as source. Moreover, a faint band with a molecular mass of ∼62 kDa, corresponding to the size of cHK heavy chain, was precipitated. When NHP was used as source mAb HK-6 precipitated a band with a molecular mass of ∼120 kDa, corresponding to the size of HK.

### Establishment of Cleaved H-kininogen Sandwich Enzyme-Linked Immunosorbent Assay

We developed a cHK specific non-competitive sandwich ELISA by selecting mAb 19-20-3 as the capture antibody. The HK/cHK specific mAb 19-31-18 was biotinylated and used as detection antibody.

**FIGURE 4:**
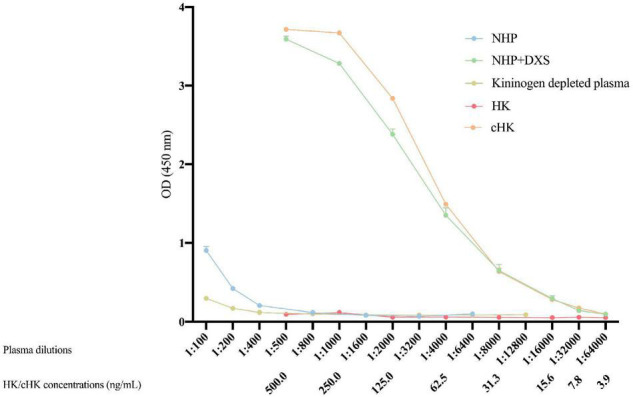
Cleaved H-kininogen specific non-competitive sandwich ELISA. cHK levels were measured by the cHK specific non-competitive ELISA using monoclonal antibody (mAb) 19-20-3 as capture antibody and biotinylated mAb 19-31-18 as detection antibody. Levels of cHK were measured in normal human plasma (NHP) *in vitro* activated with dextran sulfate (NHP + DXS). For comparison, the cHK level was measured in NHP, purified cHK, and HK in twofold dilutions. The cHK signals are given as optical density (OD) at 450 nm. Error bars indicate mean and range of a double determination.

Strong signals were obtained in both NHP + DXS and purified cHK. NHP, kininogen depleted plasma or using purified HK revealed no signals (Figure 4). Thus, a specific ELISA for detection of cHK was established using mAb 19-20-3 as capture antibody and mAb 19-31-18 as detection antibody.

### Assay Validation, Reference Interval, and Effect of Oral Contraceptives

**FIGURE 5:**
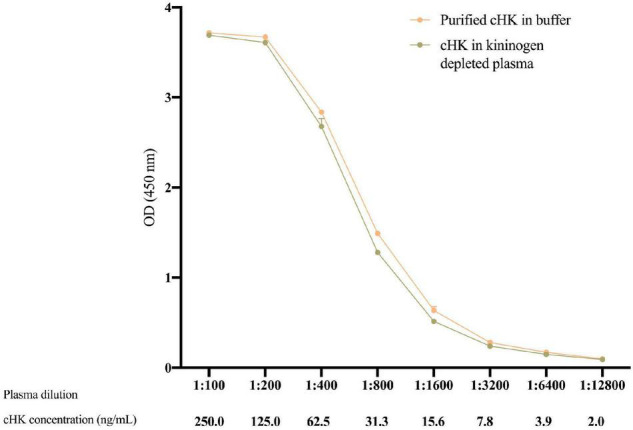
The parallelism between twofold dilutions of purified cHK diluted in buffer and cHK diluted in kininogen depleted plasma was investigated. The cHK levels are given as optical density (OD) at 450 nm. Error bars indicate mean and range of a double determination.

The validation experiments revealed an intra-assay CV of 3.6%, while the inter-assay CV was 6.0%. The limit of detection of the cHK ELISA was 1.01 ng/mL. Parallelism was observed between the calibrator curve and purified cHK in buffer, *p* = 0.98 (Figure 5).

**FIGURE 6:**
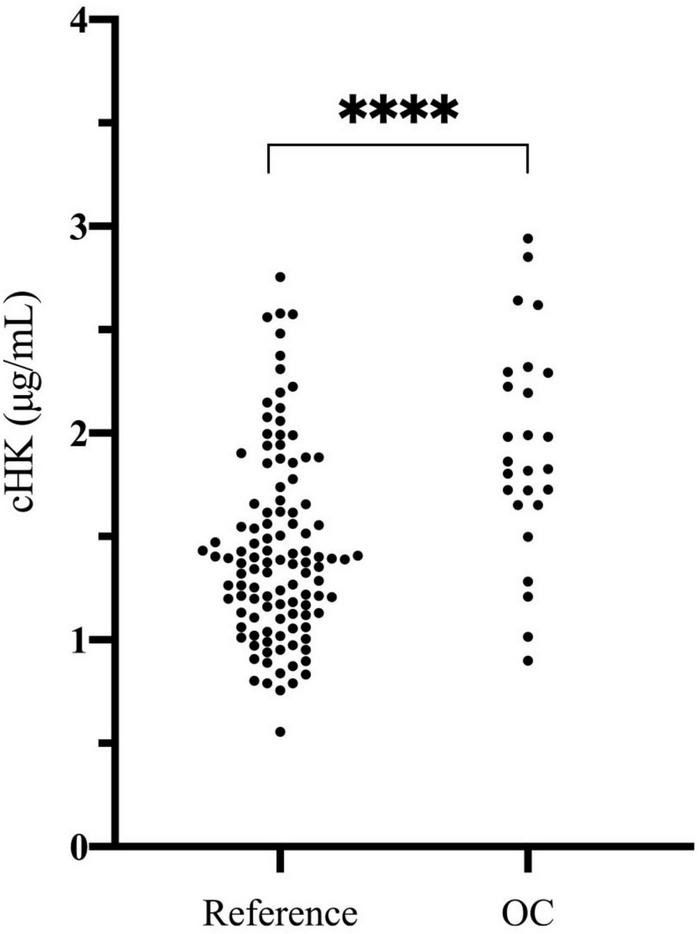
Cleaved H-kininogen levels determined in citrate stabilized plasma from a reference population of 120 healthy blood donors (60 women and 60 men) (Reference) and in citrate stabilized plasma from 26 healthy female blood donors subscribed to oral contraceptives (OC). The median plasma concentration of cHK in the reference population was 1.38 (0.82 – 2.56) μg/mL. The median plasma concentration of cHK in women using oral contraceptives was significantly higher than in the reference population [1.84 (0.94 – 2.91) μg/mL], ^****^p < 0.001.

The plasma concentration of cHK was not normally distributed (*p* < 0.001). The median concentration of cHK was not significantly different among women 1.41 (0.79 – 2.58) μg/mL and men 1.36 (0.81 – 2.47) μg/mL, *p* = 0.29. Consequently, the results were combined establishing a reference interval of 0.82 – 2.56 μg/mL with a median of 1.38 μg/mL (Figure 6).

### Evaluation of Contact Activation *ex vivo* and *in vitro*

The plasma concentration of cHK in women using OC was 1.84 (0.94 – 2.91) μg/mL, which was significantly higher than the reference population, *p* < 0.001 (Figure 6).

**FIGURE 7:**
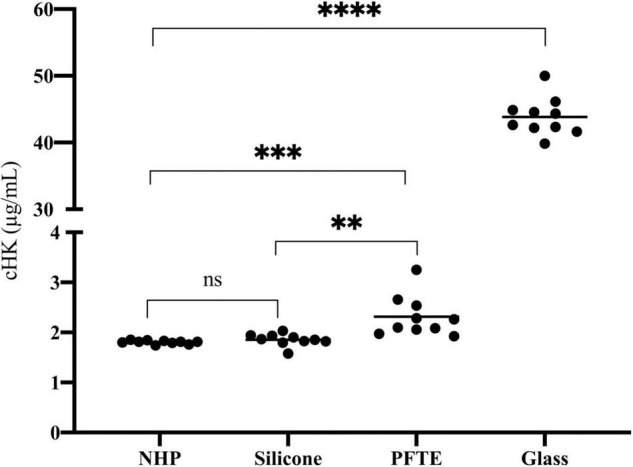
The effect of artificial surfaces on the plasma concentration of cHK. The effect of silicone (*n* = 10), polytetrafluoroethylene (PFTE) (*n* = 10) and glass (*n* = 10) on cHK concentrations in normal human plasma (NHP) after incubation for 2 h were compared to control NHP without incubation (*n* = 10). No significance (ns) Silicone compared to NHP. **p = 0.003 Silicone compared to PFTE. ^***^p = 0.001 PFTE compared to NHP. ^****^p < 0.0001 Glass compared to NHP. Horizontal lines indicate the mean levels.

We observed a significant difference in cHK between citrate plasma before incubation (time zero) and citrate plasma incubated in PFTE tubings (*p* = 0.001) and a 14-fold increase in cHK levels in citrate plasma after incubation in glass tubes, *p* < 0.0001. A significant difference in cHK was also observed between citrate plasma incubated in silicone and PFTE, *p* = 0.003 (Figure 7).

## Discussion

In this study, we have developed a panel of mAbs, called 19-20-x, toward human cHK with specificity to the end sequence of the C-terminal region of HK generated upon cleavage induced by PKa. The specificity of the mAbs were in-depth evaluated in various setups including, ELISA, SPR, and immunoprecipitation. The mAbs were confirmed to be cHK specific, without cross-reactivity with native (un-cleaved) HK. We established and validated a sandwich ELISA based on one of the cHK specific antibodies 19-20-3. We established the reference interval for cHK in human plasma and demonstrated a significantly elevated plasma concentration of cHK in women receiving OC and we demonstrated elevated levels of cHK in plasma exposed to artificial surfaces.

PKa cleaves intact HK generating cHK and three cleavage sites are known. Initially HK is cleaved at the positions Lys_362_-Arg_363_ and Arg_371_-Ser_372_, inducing release of bradykinin and yielding an intermediate cHK with a heavy chain of ∼62 kDa and a light chain of ∼56 kDa. Next, PKa cleaves cHK at position Arg_419_-Lys_420_, yielding a truncated form of HK, reducing the light chain of cHK to ∼46 kDa (15).

The cHK specific antibodies generated in this study are based on peptide immunization and a screening procedure using overlapping peptide contra cleaved peptide in parallel with intact HK and cHK. This strategy was selected to achieve mAbs specifically directed towards cHK cleaved by PKa. A direct ELISA demonstrated the specificity of the mAbs (Figure 2A). SPR analysis of the selected mAbs confirmed the specificity towards cHK (Figure 2B). SPR further characterized the selected mAb 19-20-3 demonstrating strong binding with affinity in the nanomolar range (7.5 nM) with mAb immobilized on the chip. Importantly, 19-20-3 also exhibited strict selectivity for cHK versus HK regardless whether mAb or antigen was bound on the sensor chip.

Immunoprecipitation further demonstrated the specificity of the selected capture mAb 19-20-3 showing no visible bands corresponding to intact HK when NHP + Glass and NHP was used as source (Figure 3). Moreover, a sandwich ELISA employing mAb 19-20-3 as capture antibody and mAb 19-31-18 as detection antibody revealed high signals in contact activated plasmas and purified cHK, while low signals were recorded in NHP, kininogen depleted plasma, and purified HK (Figure 4).

The reference interval of cHK in human citrate plasma was established according to international guidelines (25), i.e., from 120 healthy individuals. The concentration of cHK was comparable among men and women. The combined results, however, were not normally distributed and consequently the reference interval is given as the 5–95 percentile range of the distribution corresponding to 0.82 – 2.56 μg/ml with a median of 1.38 μg/mL. These data indicate a substantial ongoing contact activation in normal healthy individuals resulting in a significant turnover of native HK as the plasma concentration of native HK is 70–90 μg/mL.

Previous studies support that cleaved HK is a valuable marker of contact activation in clinical settings. Suffritti et al., using electrophoresis technique, and Hofman et al., using a semi-specific nanobody against cHK, demonstrated that cHK is a good indirect marker for bradykinin release in patients suffering from hereditary angioedema (7, 26). Notably, cHK is much more stable in plasma than bradykinin (27). Yamamoto-Imoto et al., using a conformational specific antibody, identified cHK as a potential biomarker for Alzheimer’s disease (28), and recent studies suggest contact factors as candidate biomarkers for diagnosis of this disorder (29).

In the current study, we evaluated the use of the cHK ELISA *ex vivo* by determination of the plasma concentration of cHK in women receiving OC. The plasma concentration of FXII and PK is increased by OC whereas the concentration of C1-inh is significantly reduced (30), suggesting an increased contact activation potential in women using OC. The developed cHK assay addressing the combined effect of OC on contact activation proteins, demonstrates an increased HK-turnover in women taking OC. Thus, the assay is suitable for evaluation of contact activation in a clinical context.

*In vitro* evaluation of plasma exposed to the biomaterials silicone and PFTE tubings as well as glass tubes was analyzed using the cHK specific assay. cHK levels in plasma incubated with PFTE tubings were significantly higher than in cHK in plasma before incubation (time zero). Plasma incubated with glass, which is known to activate the contact system significantly (31), revealed the highest cHK levels. Taken together these findings strongly suggest that the cHK assay has the capacity to detect *in vitro* activation of the contact system distinguishing between contact activation induced by various plastic materials. Thus, the assay is suitable for evaluation of the contact activating capacity of biomaterials.

Using our previously developed ELISA we demonstrated that F-α_2_M is a sensitive and specific marker of contact activation *in vitro* (12). The specificity of F-α_2_M, however, may be challenged when contact activation is assessed *ex vivo*. In clinical samples many proteases, beside those of the contact system may interact with α_2_-macroglobulin leading to formation of F-α_2_M and thus contribute to the plasma concentration of F-α_2_M. The unique specificity of the cHK catching mAb, reported here, ensures that only HK cleaved by PKa *ex vivo* contributes to the plasma concentration of cHK.

## Conclusion

In conclusion, our findings demonstrate that cHK is a sensitive and specific marker of contact activation both *in vitro* and *ex vivo*. The cHK ELISA could be an important tool in the diagnosis of diseases, assessment of treatment efficacy and as a prognostic indicator in diseases where contact activation contributes to an inflammatory response. Furthermore, the cHK ELISA can be useful for *in vitro* investigations of the plasma compatibility of BCB in relation to the contact system.

## Data Availability Statement

The raw data supporting the conclusions of this article will be made available by the authors, without undue reservation.

## Ethics Statement

Ethical review and approval were not required for the study on human participants in accordance with the local legislation and institutional requirements. The patients/participants provided their written informed consent to participate in this study. The animal study was reviewed and approved by the Animal Experiments Inspectorate Stationsparken 31-33 DK 2600 Glostrup Tel.+45 72 27 69 00 Licens nr. 2020-15-0201-00471.

## Author Contributions

YP, STDP, KP, and JG performed the analyses. YP, JBG, and JJS designed and directed the research. YP, STDP, and JJS wrote the manuscript. All authors contributed to the article and approved the submitted version.

## Conflict of Interest

The authors declare that the research was conducted in the absence of any commercial or financial relationships that could be construed as a potential conflict of interest.

## Publisher’s Note

All claims expressed in this article are solely those of the authors and do not necessarily represent those of their affiliated organizations, or those of the publisher, the editors and the reviewers. Any product that may be evaluated in this article, or claim that may be made by its manufacturer, is not guaranteed or endorsed by the publisher.
